# Two new species of oribatid mites of the genus *Truncozetes* (Acari, Oribatida, Epactozetidae) from Ecuador

**DOI:** 10.3897/zookeys.303.5309

**Published:** 2013-05-21

**Authors:** Sergey G. Ermilov, Dorothee Sandmann, Franca Marian, Mark Maraun

**Affiliations:** 1 Tyumen State University, Tyumen, Russia; 2 Georg-August-University Göttingen, J.F. Blumenbach Institute of Zoology and Anthropology, Göttingen, Germany

**Keywords:** Oribatid mites, Epactozetidae, *Truncozetes*, new species, key, Ecuador

## Abstract

Two new oribatid mite species of the genus *Truncozetes* (Oribatida, Epactozetidae), *Truncozetes ecuadoriensis* **sp. n.** and *Truncozetes monodactylus* **sp. n.**, are described from the Ecuadorian soils. The morphology of the gnathosoma and the legs is presented in detail for the first time for the species of *Truncozetes*. An identification key to all known species of the family Epactozetidae is given.

## Introduction

Epactozetidaeis a small oribatid mite family of the superfamily Achipterioidea (Acari, Oribatida), comprising of two genera (*Epactozetes* Grandjean, 1930, *Truncozetes* Balogh and Mahunka, 1969) and five species, which are distributed in the Neotropical region.

*Epactozetes* is a genus that was proposed by Grandjean (1930) with *Epactozetes imitator* Grandjean, 1930 as type species. Currently, this genus comprises two species: *Epactozetes imitator* Grandjean, 1930 (see Grandjean 1930) and *Epactozetes setosus* Balogh and Mahunka, 1969 (see Balogh and Mahunka 1969b). The main diagnostic characters of this genusare (summarized from Balogh and Balogh 1988, 1992; with our opinions): lamellae as long as prodorsum, fused or connected medio-anteriorly; genital plates with five pairs of setae; leg tarsi with three claws.

*Truncozetes* is a genus that was proposed by Balogh and Mahunka (1969a) with *Truncozetes mucronatus* Balogh and Mahunka, 1969 as type species. Currently, this genus comprises three species: *Truncozetes mucronatus* Balogh and Mahunka, 1969 (see Balogh and Mahunka 1969a), *Truncozetes rugosus* Mahunka, 1998 (see Mahunka 1998) and *Truncozetes sturmi* Balogh, 1984 (see Balogh 1984). The main diagnostic characters of this genusare (summarized from Balogh and Mahunka 1969a; Balogh and Balogh 1988, 1992; with our opinions): lamellae shorter than prodorsum, well separated, connected by translamella; notogaster with large posterior tubercle; genital plates with five to six pairs of setae; leg tarsi with one or three claws (one species with monodactylous legs I and tridactylous legs II–IV).

During taxonomic identification of the Ecuadorian oribatid mite fauna, we discovered two new epactozetid species belonging to the genus *Truncozetes*. The main purpose of this paper is to describe and illustrate these species under the names *Truncozetes ecuadoriensis* sp. n.and *Truncozetes monodactylus* sp. n. The morphology of the gnathosoma and the legs is presented in detail for the first time for the species of the genus *Truncozetes*.

An identification key to all known species of the family Epactozetidae is provided.

## Materials and methods

Specimens were mounted in lactic acid on temporary cavity slides for measurement and illustration. Body length was measured from the side, i.e. from the tip of the rostrum to the posterior edge of the ventral plate. Notogastral width refers to the maximum width in dorsal aspect. Lengths of body setae were measured from the lateral side. All body measurements are given in micrometers (μm). Formulae for leg setation are given in parentheses according to the sequence trochanter–femur–genu–tibia–tarsus (famulus included). Formulae for leg solenidia are given in square brackets according to the sequence genu–tibia–tarsus.

General terminology used in this paper follows that summarized by Norton and Behan-Pelletier (2009).

## Descriptions of new species

### Truncozetes ecuadoriensis sp. n.

urn:lsid:zoobank.org:act:DF076204-3191-4390-89C5-D356720287A0

http://species-id.net/wiki/Truncozetes_ecuadoriensis

Figs 1, 2 , 5 –17

#### Diagnosis.

Body size 315–332 × 215–232. Translamella thin, straight. Sensilli with weakly barbed elongate-oval head. Five pairs of genital and two pairs of anal setae present. Leg tarsus I monodactylous, leg tarsi II–IV tridactylous.

#### Description.

*Measurements*. Body length: 315 (holotype), 315, 332 (two paratypes); notogaster width: 215 (holotype), 215, 232 (two paratypes).

*Integument*. Body color brown. Surface covered by cerotegumental microgranules (visible under high magnification). Foveolae distinct, larger on pteromorphs (diameter up to 12).

*Prodorsum*. Rostrum widely rounded. Lamellae with lateral point anteriorly. Translamella very thin, straight. Rostral setae (*ro*) of medium size (41–45), setiform, barbed. Lamellar (*le*, 6–8) and interlamellar (*in*, 2–4) setae short, thin, smooth. Sensilli (*ss*, 53–61) with short stalk and weakly barbed elongate-oval, head. Exobothridial setae and their alveoli absent. Tutotia (*tu*) knife-form, with long and sharp cusps, reaching insertions of rostral setae.

*Notogaster*. Weakly concave posteriorly. Posterior tubercle (*tbp*) poorly developed. Ten pairs of short (12–16), thin, smooth notogastral setae. Three pairs of small sacculi: *Sa* inserted antero-medially to setae *la*; *S1* – antero-medially to setae *h*_2_; *S2*– antero-laterally to setae *h*_1_. Lyrifissures *ia* located on pteromorphs, but poorly visible; *im*– antero-laterally to setae *h*_3_; *ip* – postero-laterally to sacculi *S2*; *ih* and *ips* located in lateral positions. Opisthonotal gland openings not found.

*Gnathosoma*. Subcapitulum longer than wide (77 × 61). Subcapitular (*h*, *m*, *a*) and adoral (*or*_1_, *or*_2_) setae similar in length (12) setiform, smooth. Palps (61) with setation 0–2–1–3–9 (+ω). Solenidion (ω) thickened, straight, attached with eupathidium (*acm*). Chelicerae (77) with one setiform, barbed seta (*cha*, 24); possible *chb* also present, but we found only their alveolus in dissected specimen. Trägårdh’s organ (Tg) long, elongate conical.

*Epimeral and lateral podosomal regions*. Apodemal border 4 (*bo4*) complete, wide, brownish. Epimeral setal formula: 3–1–2–2. Epimeral setae short, setiform, smooth; *1a*, *3a* (2) shorter than *2a* (8), *1b*, *1c*, *3b*, *4a*, *4b* (12–16). Discidia (*dis*) triangular. Circumpedal carinae (*cp*) distinct.

*Anogenital region*. Five pairs of genital (*g*_1_–*g*_5_, 8), one pair of aggenital (*ag*, 2–4), two pairs of anal (*an*_1_, *an*_2_, 2–4) and two pairs of adanal (*ad*_1_, *ad*_2_, 2–4) setae short, setiform, thin, smooth. Lyrifissures *iad* located in paraanal position.

*Legs*. Tarsus I with one claw, tarsi II–IV with three claws. Dorsal side of tarsus I and dorso-proximal part of tibia IV with strong thorn (*t*); antero-ventral side of genu I with small thorn; ventral side of tarsus I and tibia I with large tubercles (*tb*). Formulae of leg setation and solenidia: I (1–5–3–4–20) [1–2–2], II (1–5–2–3–15) [1–1–2], III (1–2–1–2–15) [1–1–0], IV (0–2–1–2–12) [0–1–0]; homology of setae and solenidia indicated in Table 1. Famulus (*e*) thin, straight, inserted anteriorly to thorn. Setae barbed (except smooth *p* and *s* on tarsus I). Solenidia ω_1_ on tarsus I, ω_1_, ω_2_o n tarsus II, σ on genua III thickened, blunt-ended, other solenidia setiform.

**Figures 1–4.**
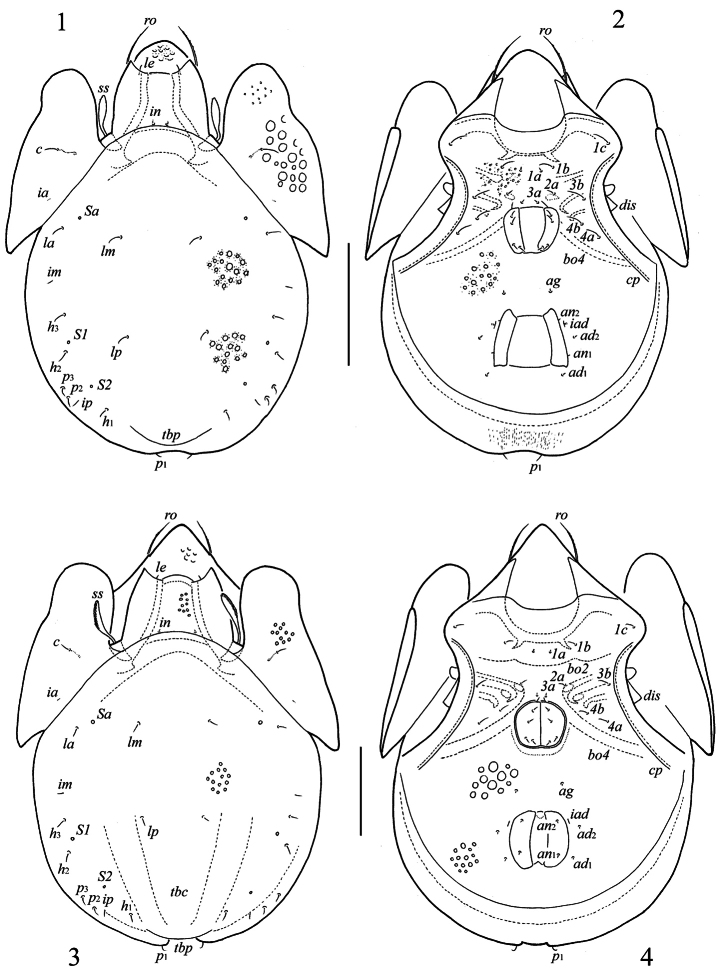
*Truncozetes ecuadoriensis* sp. n. (1, 2) and *Truncozetes monodactylus* sp. n. (3, 4), adults. **1, 3** body dorsally **2, 4** body ventrally (gnathosoma and legs not illustrated). Scale bars: (1, 2) 100 μm, (3, 4) 50 μm. Abbreviations in text.

**Figures 5–13.**
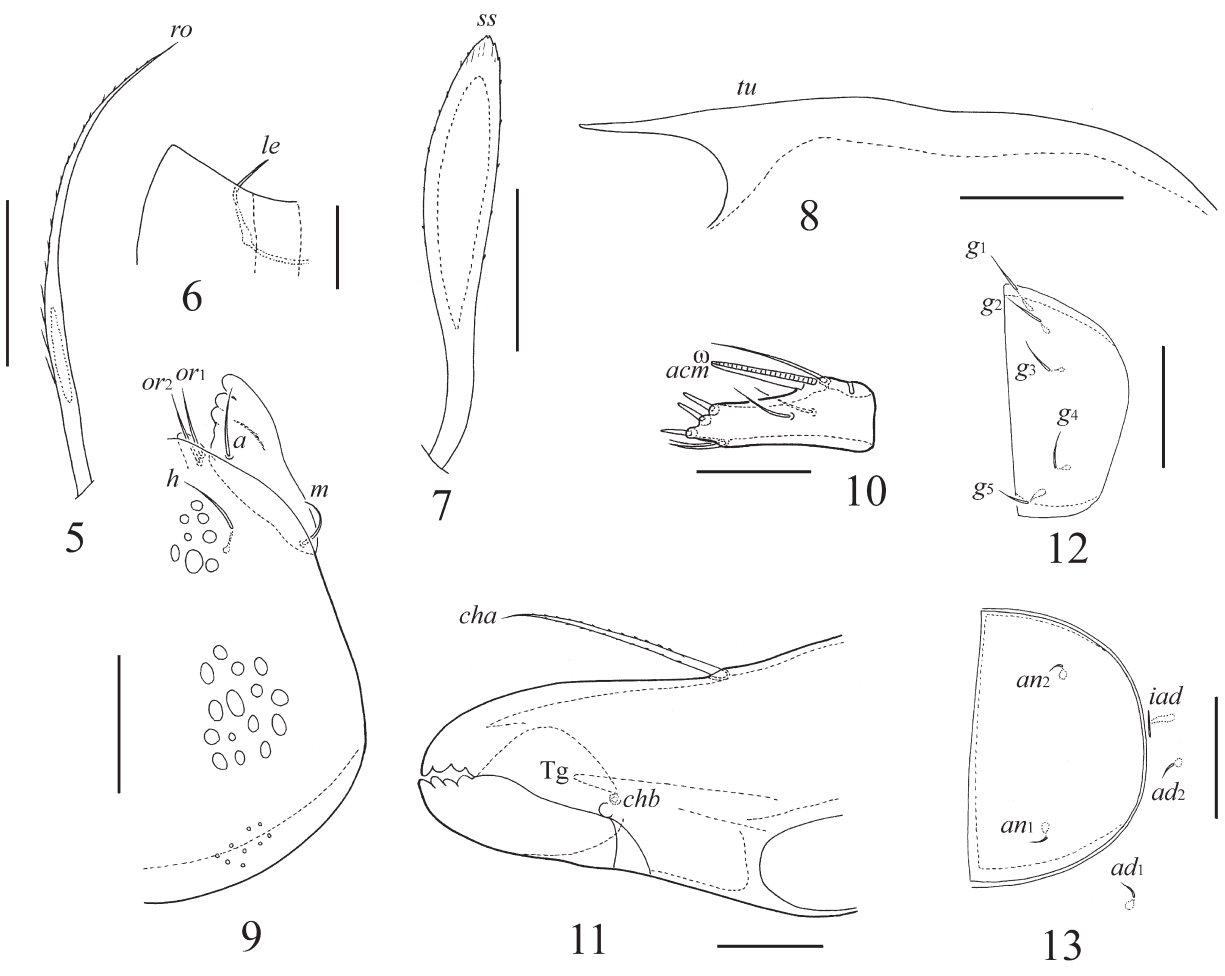
*Truncozetes ecuadoriensis* sp. n., adult. **5** rostral seta **6** lamellar seta and anterior part of lamella dorsally **7** sensillus **8** tutorium **9** subcapitulum ventrally, left part **10** palptarsus laterally **11** anterior part of chelicera **12** genital plate, left **13** anal plate, left. Scale bars: (**5, 7–9, 12, 13**) 20 μm, (**6, 10, 11**) 10 μm. Abbreviations in text.

**Figures 14–17.**
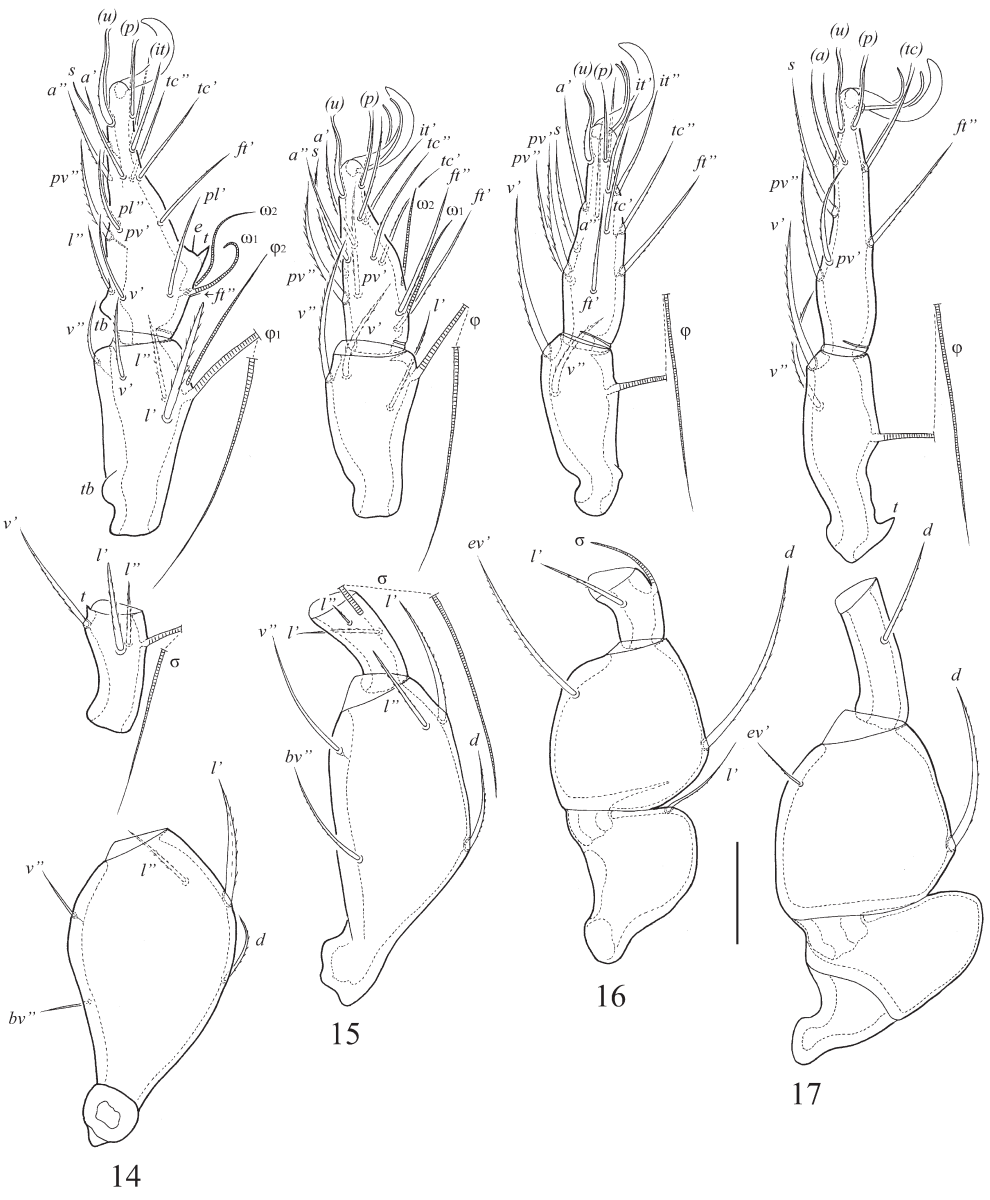
*Truncozetes ecuadoriensis* sp. n., adult. **14** leg I (without trochanter), right, paraxial view **15** leg II (without trochanter), left, antiaxial view **16** leg III, right, antiaxial view **17** leg IV, right, antiaxial view. Scale bar: 20 μm. Abbreviations in text.

**Table 1.** Leg setation and solenidia of *Truncozetes ecuadoriensis* sp. n. (same for *Truncozetes monodactylus* sp. n.)

**Leg**	**Trochanter**	**Femur**	**Genu**	**Tibia**	**Tarsus**
I	*v’*	*d, (l), bv’’, v’’*	*(l), v’*,σ	*(l), (v)*,φ_1_,φ_2_	*(ft), (tc), (it), (p), (u), (a), s, (pv), v’, (pl), l’’, e*,ω_1_,ω_2_
II	*v’*	*d, (l), bv’’, v’’*	*(l)*,σ	*l’, (v)*, φ	*(ft), (tc), (it), (p), (u), (a), s, (pv)*,ω_1_,ω_2_
III	*l’*	*d, ev’*	*l’*,σ	*(v)*,φ	*(ft), (tc), (it), (p), (u), (a), s, (pv)*
IV	-	*d, ev’*	*d*	*(v)*, φ	*ft’’, (tc), (p), (u), (a), s, (pv)*

Roman letters refer to normal setae (*e* to famulus), Greek letters to Solenidia. Single prime (*‘*) marks setae on anterior and double prime (*“*) setae on posterior side of the given leg segment. Parentheses refer to a pair of setae.

#### Material examined.

Holotype (male), two paratypes (male, female): Ecuador, 3°58'S, 79°50'W, Estación Científica San Francisco, 2000 m a.s.l., upper organic soil layer in mostly undisturbed rain forest, 01.04.2009, collected by F. Marian and D. Sandmann.

#### Type deposition.

The holotype (in alcohol) is deposited in the collection of the Zoological Institute of the Russian Academy of Sciences, St. Petersburg, Russia; one paratype (in alcohol) is deposited in the collection of the Siberian Zoological Museum, Novosibirsk, Russia; one paratype (dissected) is in the personal collection of the first author.

#### Etymology.

The specific name “*ecuadoriensis*” refers to the country of origin, Ecuador.

#### Remarks.

The new species is clearly distinguishable from other known species of the genus *Truncozetes* by the different number of leg claws (leg I monodactylous, legs II–IV tridactylous versus all legs monodactylous or tridactylous). Additional distinctive characters of a new species with the other species of the genus can be found in the identification key given below.

### Truncozetes monodactylus sp. n.

urn:lsid:zoobank.org:act:C92C81FB-B822-44D3-899B-056490CC1C47

http://species-id.net/wiki/Truncozetes_monodactylus

Figs 3, 4

#### Diagnosis.

Body size 232 × 166. Translamella concave medially. Sensilli with lanceolate head densely ciliated on dorsal side. Dorso-central part of notogaster with large tubercle. Five pairs of genital and two pairs of anal setae. All leg tarsi monodactylous.

#### Description.

*Measurements*. Body length: 232 (holotype and paratype), 166 (holotype and paratype).

*Integument*. Body color brown. Surface covered by cerotegumental microgranules (visible under high magnification). Foveolae distinct, small (diameter up to 6).

*Prodorsum*. Rostrum narrowly rounded. Lamellae with lateral point anteriorly. Translamella thick, concave medially. Rostral setae of medium size (24), setiform, barbed. Lamellar (4) and interlamellar (2) setae minute. Sensilli (32) with short stalk and elongate-oval head, which is densely ciliate on dorsal side. Exobothridial setae and their alveoli absent. Tutotia knife-form, reaching insertions of rostral setae.

*Notogaster*. Concave posteriorly. Dorso-central part convex, with elongate hump-like tubercle (*tbc*). Posterior tubercle (*tbp*) well developed. Ten pairs of short (8–12), thin, smooth notogastral setae present. Three pairs of small sacculi visible, but *S2* poorly visible. Position of lyrifissures asin *Truncozetes ecuadoriensis* sp. n. Opisthonotal gland openings not found.

*Gnathosoma*. Similar to *Truncozetes ecuadoriensis* sp. n.

*Epimeral and lateral podosomal regions*. Apodemal borders 2 (*bo2*) and 4 (*bo4*) wide, fused medially, brownish. Epimeral setal formula: 3–1–2–2. Epimeral setae short, setiform, smooth; *1a*, *3a* (2) shorter than *2a* (4), *1b*, *1c*, *3b*, *4a*, *4b* (8). Discidia triangular. Circumpedal carinae distinct.

*Anogenital region*. Five pairs of genital (4), one pair of aggenital (2), two pairs of anal (2) and two pairs of adanal (2) setae short. Lyrifissures *iad* located in paraanal position.

*Legs*. Similar to *Truncozetes ecuadoriensis* sp. n., but all tarsi with one strong claw.

#### Material examined.

Holotype (female), one paratype (female): Ecuador, 3°70'S, 78°58'W, Bombuscaro, Podocarpus National Park, 1050 m a.s.l., upper organic soil layer in mostly undisturbed rain forest, 01.04.2009, collected by F. Marian and D. Sandmann.

#### Type deposition.

The holotype (in alcohol) is deposited in the collection of the Zoological Institute of the Russian Academy of Sciences, St. Petersburg, Russia; one paratype (dissected) is in the personal collection of the first author.

#### Etymology.

The specific name “*monodactylus*” refers to the one claw on all leg tarsi.

#### Remarks.

The new species is clearly distinguishable from other known species of the genus *Truncozetes* by the monodactylous legs (versus all legs tridactylous or leg I monodactylous, legs II–IV tridactylous). Additional distinctive characters of this species from other species of the genus can be found in the identification key given below.

##### Key to known species of Epactozetidae

**Table:** 

1	Lamellae shorter than prodorsum, well separated, connected by translamella	2 (genus *Truncozetes*)
–	Lamellae as long as prodorsum, fused or connected medio-anteriorly	6 (genus *Epactozetes*)
2	All leg tarsi with one claw; apodemal borders II and IV fused medially; body size: 232 × 166	*Truncozetes monodactylus* sp. n. (Distribution: Ecuador)
–	All leg tarsi with three claws or only leg tarsus I with one claw; apodemal borders II and IV not fused medially	3
3	Leg tarsus I with one claw, leg tarsi II–IV with three claws; genital plates with five pairs of setae; body size: 315–332 × 215–232	*Truncozetes ecuadoriensis* sp. n. (Distribution: Ecuador)
–	All leg tarsi with three claws; genital plates with six pairs of setae	4
4	Dorsal notogastral setae *lm* and *lp* inserted in lateral position of notogaster, approximately in one longitudinal row with setae *la* and *h*_3_; distal part of sensillus dark; body size: 233–244 × 171–176	*Truncozetes rugosus* Mahunka, 1998 (Distribution: Antilles)
–	Dorsal notogastral setae *lm* and *lp* inserted in dorsal position of notogaster; distal part of sensillus not dark	5
5	Epimeral region with distinct longitudinal stria; sensillar heads densely barbed; body size: 228 × 168	*Truncozetes mucronatus* Balogh & Mahunka, 1969 (Distribution: Neotropical region)
–	Epimeral region without longitudinal stria; sensillar heads smooth; body size: 308–336 × 176–185	*Truncozetes sturmi* Balogh, 1984 (Distribution: Neotropical region)
6	Lamellae fused medio-anteriorly; notogastral setae visible; body size: 235–270 × 180–235	*Epactozetes setosus* Balogh & Mahunka, 1969 (Distribution: Neotropical region)
–	Lamellae connected medio-anteriorly; notogastral setae not visible; body size: 210–235 × 160	*Epactozetes imitator* Grandjean, 1930 (Distribution: Central America)

## Supplementary Material

XML Treatment for Truncozetes ecuadoriensis

XML Treatment for Truncozetes monodactylus
